# Exploring the spatio-temporal functional connectome in PTSD: evidence of static and dynamic network alterations

**DOI:** 10.1186/s12888-026-08500-4

**Published:** 2026-08-06

**Authors:** Jing Shang, Minlan Yuan, Xian Mo, Junran Zhang, Changjian Qiu, Su Lui, Hongru Zhu, Wei Zhang

**Affiliations:** 1 Department of Psychiatry, The First Affiliated Hospital of Soochow University, Suzhou, Jiangsu China; 2 Mental Health Center, National Center for Mental Disorders, West China Hospital, Sichuan University, Chengdu, China; 3 Huaxi Brain Research Center, West China Hospital, Sichuan University, Chengdu, China; 4 West China Biomedical Big Data Center, West China Hospital, Sichuan University, Chengdu, China; 5 College of Electrical Engineering, Sichuan University, Chengdu, Sichuan China; 6 Huaxi MR Research Center (HMRRC), Department of Radiology, West China Hospital, Sichuan University, Chengdu, Sichuan China; 7 Med-X Center for Informatics, Sichuan University, Chengdu, Sichuan 610041 China

**Keywords:** Post-traumatic stress disorder, Dynamic functional connectivity, Intrinsic functional connectivity, Resting-state functional magnetic resonance imaging

## Abstract

**Background:**

Few studies with substantial sample size have investigated the neural pathophysiology of post-traumatic stress disorder (PTSD). We aimed to characterize both static and dynamic functional network connectivity (dFNC) in a large, homogeneous, treatment-naïve PTSD cohort.

**Methods:**

Resting-state fMRI data from 70 PTSD patients and 68 demographically matched, trauma-exposed control (TEC) participants were analyzed using independent component analysis (ICA) and a sliding-window k-means clustering approach.

**Results:**

PTSD patients exhibited widespread alterations in both static intrinsic functional connectivity (iFC) and dFNC, spanning frontoparietal, subcortical, and visual networks. Variations in static iFC were significantly correlated with symptom severity (primarily measured by Clinician-Administered Posttraumatic Stress Disorder Scale and Hamilton Anxiety Rating Scale scores). Significant group differences in dFNC connectivity patterns were identified in six of the seven dynamic states (States 1–3 and 5–7), while no significant between-group differences were observed in the temporal properties (fractional occupancy, mean dwell time, and transition rates) across any of the states.

**Conclusion:**

These convergent results provide additional evidence for iFC and dFNC dysregulation in PTSD, suggesting that network-level abnormalities particularly within frontoparietal, primary sensory and subcortical systems may contribute to the neural mechanisms of the disorder.

**Clinical trial number:**

Not applicable.

**Supplementary Information:**

The online version contains supplementary material available at https://doi.org/10.1186/s12888-026-08500-4.

## Introduction

Posttraumatic stress disorder (PTSD) is a debilitating psychiatric condition affecting individuals exposed to traumatic events, with a constellation of symptoms including intrusive re-experiencing, persistent avoidance of trauma-related stimuli, detrimental changes in cognition and emotional state, and hypervigilance [1]. Despite its clinical significance, PTSD exhibits substantial heterogeneity, complicating diagnosis and treatment [2]. Research has identified distinct symptom clusters, such as fear-based (e.g., re-experiencing) and dysphoric (e.g., emotional numbing) profiles, linked to unique brain connectivity patterns [2]. A dissociative subtype (PTSD + DS) has also been recognized, marked by depersonalization/derealization and distinct neurophysiology [3, 4]. This diversity highlights the need for neurobiological markers to complement symptom-based diagnostics and enable personalized treatments [5].

Neuroimaging research has established PTSD as fundamentally a disorder of brain dysconnectivity, characterized by aberrant intercommunication within and between extensive neural networks [6, 7]. Static functional network connectivity (sFNC) studies using resting-state fMRI have revealed a canonical model of PTSD pathophysiology centered on dysregulation of three fundamental networks: the default mode network (DMN), salience network (SN), and central executive network (CEN) [8]. The DMN shows reduced internal connectivity, potentially underlying the fragmented sense of self and pathological integration of traumatic memories [9, 10]. The SN, anchored by the anterior cingulate cortex and insula, exhibits hyperconnectivity with sensory regions, reflecting hypervigilance and enhanced threat detection [11]. The CEN demonstrates weakened connectivity, particularly in prefrontal regions, corresponding to impaired cognitive control and emotion regulation [12]. These network alterations collectively contribute to PTSD’s core symptoms, yet static approaches assume fixed connectivity patterns throughout scanning sessions, potentially obscuring crucial temporal dynamics.

However, static functional connectivity (sFC) approaches assume stationary brain organization, obscuring rich temporal dynamics. Recent evidence suggests that psychiatric disorders may be characterized by alterations in dynamic functional network connectivity (dFNC) - the moment-to-moment reconfiguration of brain networks [13]. Despite this promise, dFNC findings in PTSD remain mixed and limited, with studies reporting both increased variability [14]and reduced flexibility [8], highlighting critical gaps: (1) lack of well-characterized, homogeneous samples; (2) limited whole-brain, data-driven approaches; and (3) unclear relationships between dynamic states and clinical symptoms.

The present study addresses these gaps by investigating dynamic network reconfiguration in treatment-naive, non-comorbid adults with PTSD versus demographically matched trauma-exposed controls (TECs). We combine data-driven ICA to define intrinsic connectivity networks with sliding-window dFNC and k-means clustering to comprehensively characterize network abnormalities. We hypothesized that: (1) PTSD would show altered sFNC within and between cognitive (FPN, DMN), salience, and sensory/subcortical networks, correlating with symptom severity; and (2) PTSD would exhibit disrupted dynamic connectivity properties, specifically aberrant connectivity patterns within recurrent dFNC states involving interactions between higher-order cognitive and sensory-affective networks. This approach aims to reveal the moment-to-moment brain dynamics underlying PTSD’s complex symptomatology.

## Methods

### Participants

The study enrolled survivors from a heavily impacted area near the epicenter of the 2008 Wenchuan earthquake. Prior to study initiation, ethical approval was procured from the Medical Research Ethics Committee of West China Hospital, Sichuan University, and written informed consent was acquired from all participants. MRI scans were acquired between 2012 and 2015, representing a chronic stage of PTSD. We employed mean FD_Jenkinson [15] as our primary metric for assessing head motion. Participants were excluded if their mean FD_Jenkinson exceeded 0.5 mm. Following the exclusion of 13 subjects due to excessive head movement, the final dataset comprised whole-brain resting-state fMRI scans from 68 TECs and 70 adults with PTSD. Symptom severity was further assessed with Clinician-Administered Posttraumatic Stress Disorder Scale (CAPS), the Hamilton Rating Scale for Depression-17 (HAMD-17) and the Hamilton Anxiety Rating Scale (HAMA). The “non-comorbid” status was determined via the Structured Clinical Interview for DSM-5 (SCID-5) conducted by two experienced psychiatrists. While participants exhibited elevated HAMD and HAMA scores, they did not meet the full formal diagnostic criteria for Major Depressive Disorder (MDD) or Generalized Anxiety Disorder (GAD). These standardized scales are extensively utilized for the psychometric evaluation of PTSD patients in our previous research, providing comprehensive assessment of post-traumatic symptom severity, depression, and anxiety, higher scores indicating greater severity [16, 17]. The study procedures adhered strictly to the principles of the Declaration of Helsinki.

### MRI acquisition

Resting-state and structural fMRI were obtained at the Brain Imaging Center of Southwest University utilizing a 3.0T Siemens Tim Trio MRI system (Siemens Trio, Erlangen, Germany) equipped with a 12-channel phased array head coil. During the resting-state fMRI scanning, the participants were fitted with soft ear plugs and instructed to relax with their eyes closed; without falling asleep; and without directed, systematic thought, lasting 6 min and 48 s. The rs-fMRI scans encompassed a total of 205 volumes. Functional echo-planar images for the acquisition of resting-state data were obtained utilizing the subsequent parameters: Repetition time (TR) = 2000 ms, echo time (TE) = 30 ms, flip angle = 90°, field of view (FOV) = 240 × 240 mm^2^, 33 axial slices, slice thickness = 5.0 mm, acquisition matrix = 64 × 64, and voxel size = 3.75 × 3.75 × 5.0 mm^3^, resulting in a total of 205 volumes for each participant. High-resolution T1-weighted structural images were acquired for each participant, with the following parameters: 176 slices with thickness = 1 mm, acquisition matrix = 256 × 256, voxel size = 1 × 1 × 1 mm^3^, TR = 1900 ms, TE = 2.26 ms, and flip angle = 9°, yielding an in-plane resolution of 0.94 × 0.94 mm^2^.

### Preprocessing

Preprocessing of rs-fMRI data was performed utilizing the Statistical Parametric Mapping program (SPM12 v7771; http://www.fil.ion.ucl.ac.uk/spm/) and the Data Processing and Analysis for Brain Imaging toolbox (DPABI v6.1; http://www.rfmri.org/dpabi/) within MATLAB R2022b (MathWorks, Inc., Natick, MA). The preprocessing included the following steps: realignment to correct for head motion, co-registration of structural and functional images, and transformation to the Montreal Neurological Institute (MNI) standard space. Nuisance signals, comprising head motion (six motion parameters and their derivatives), low-frequency drift (linear, quadratic, and cubic trends), and signals from white matter (WM) and cerebrospinal fluid (CSF) voxels, were regressed away. Finally, spatial smoothing was applied with a 6-mm full-width-at-half-maximum (FWHM) Gaussian kernel.

### Group independent component analysis

Following data preprocessing, the resting-state data of all subjects were analyzed by spatial independent component analysis utilizing the GIFT toolbox (v4.0c, http://icatb.sourceforge.net) to generate intrinsic connectivity networks. A two-level data reduction strategy was employed: one at the subject-specific level and another at the group level. PCA was first employed to compress subject-specific data to 30 main components. Then data were partitioned into 20 group independent components using the expectation-maximization approach to enhance group variability. The reliability of the ICA decomposition was verified using ICASSO, with components retained only if they demonstrated a mean intra-cluster similarity value > 0.8 [18]. Subject-level spatial maps and time courses were subsequently generated using GICA back-reconstruction.

Components underwent manual validation based on spatial and spectral criteria. Spatially, we confirmed that peak activation coordinates were located within gray matter, indicating minimal overlap with blood vessels, ventricles, and other sources of artifactual signals [19]. Spectrally, components were required to exhibit a power spectrum dominated by low-frequency fluctuations, with ratios of power between 0.10 Hz to 0.15–0.25 Hz.

### Univariate SPM analysis

The SPSS 26.0 software package (IBM Corp., Armonk, NY, USA) was used for statistical analysis to calculate basic information, including age and clinical variables. Continuous data are shown as the mean ± SD. Two independent-sample t-tests with SPM12 were performed between PTSD and TEC groups independent of age and gender effect to assess intrinsic network connectivity (ICN).

The spatial maps of the selected independent components were statistically evaluated using voxel-wise one-sample t-tests on the participants’ z-maps in SPM12 (*p* < 0.001, uncorrected). For the group comparison, the resulting statistical maps from the one-sample t-tests were employed as inclusive masks to constrain the search volume for the subsequent two-sample t-tests. These two-sample t-tests, controlling for the effects of age and gender, were first thresholded at a cluster-defining threshold of *p* < 0.001 at the voxel level. The resulting clusters were then considered significant only if they survived a family-wise error (FWE) correction of *p* < 0.05 at the cluster level.

We also employed a typical, univariate approach to investigate voxel-wise relationships between the clinical severity (CAPS, HAMD, HAMA) and the ICN strength. This procedure was implemented by a multiple regression model in the SPM12 program to identify voxels within the ICN maps where connectivity was significantly associated with the clinical scores. 15 (ICNs) x 3 (Scales) x2 (positive/negative) = 90 voxel-wise Generalized Linear Mode (GLM) tests were performed. Age and gender were considered as nuisance variables. Correction for multiple comparisons was conducted with the SPM’s nonstationary cluster-level family-wise error (FWE) method. The resulting statistical maps were thresholded at *P* = 0.001 and a corrected cluster significance of *P* < 0.05.

### Dynamic functional network connectivity (dFNC)

To elucidate the dynamic aspects of FNC, the temporal dFNC toolkit within the GIFT software was employed. The sliding window method was utilized to compute dFNC through a tapered sliding window performed with the Dynamic FNC Toolbox (dFNC v1.0a) in GIFT. The resting-state time series data were segmented into a tapered sliding window (30 TRs, 60 s) which were then convolved with a Gaussian function (*r* = 3 TR), and dynamic connectivity matrices were computed using Fisher z-transformed correlations. In each window, pairwise Fisher z-transformed r values were utilized to compute dFNC. To derive the dFNC-var for each participant, the variance (r^2^) was computed across the temporal dimension of dFNC matrices to measure the temporal variability in the strength of functional network connectivity. The k-means clustering approach was then utilized to assess the persistent functional connectivity patterns (i.e., dFNC-state). The L1 distance (City-block distance) was employed to assess the similarities among the dFNC matrices. For the functional state clustering, we employed the k-means algorithm with the City-block distance metric to enhance robustness against outliers. To ensure a global optimum, the clustering process was repeated 150 times with random initializations, with a maximum of 150 iterations per run. The optimal number of clusters (k) was determined by the Elbow Criterion integrated within the GIFT toolbox, finally established as 7. The k-means algorithm established a correspondence between cluster membership and time point for each subject. Time intervals associated with each state were segmented for each individual. A representative dFNC matrix for each state (dFNC-state) was computed for each participant by averaging the dFNC matrices corresponding to the same state for subsequent analysis [20].

We seek to identify dFNC differences between TECs and PTSD for each state individually. A two-sample t-test was conducted for each dFNC, specifically for each element in the dFNC matrix, within a designated state. For each state identified, a representative dFNC matrix was generated for every individual. However, as not all participants transitioned into every state, the subsequent analysis was conducted using the available set of subject-specific, state-wise dFNC matrices to conduct 105 t-tests in each state, since there are 15 RSNs, resulting in 15 ∗ 14/2 = 105 dFNC connectivity values. The False Discovery Rate (FDR) correction was applied to control for multiple comparisons, with the significance threshold set at *p* < 0.05. A collection of 105 t-values from each state were preserved for future reference. Several key temporal features were calculated, including: (1) the fraction of time (FT), denoting the percentage of each state among the seven states, (2) mean dwell time (MDT), indicating the average duration per subject spent in a specific state, and (3) number of transitions (NT), representing the frequency of state transitions by a subject. The statistical module of the dfnc_toolbox and GRETNA [21] calculated and visualized the distribution of subjects across the identified seven states, as well as the characteristic dFNC values for each state.

## Results

### Demographic and clinical characteristics

Demographic information and clinical evaluation outcomes were represented in Table 1. No substantial differences were observed between the groups regarding age (*p* = 0.178), gender (*p* = 0.217) and mean FD (*p* = 0.764). The CAPS has emerged as one of the most prevalent structured interviews for PTSD and is a widely recognized criterion tool for evaluating PTSD diagnostic status and symptom intensity [22]. The CAPS scores were significantly elevated in PTSD patients (CAPS = 70.94 ± 21.56) compared to TECs (CAPS = 9.28 ± 8.46; *P* < 0.01; t-test). The HAMD-17 and HAMA scores were selected to assess depressed and anxious symptoms separately [23]. The HAMD (12.5 ± 6.10) and HAMA (15.26 ± 7.87) scores of PTSD patients were considerably elevated compared to the TECs (HAMD = 3.38 ± 2.97, HAMA = 3.79 ± 4.09, *p* < 0.01; t-test). The CAPS total scores were recorded for 69 persons with PTSD and 68 trauma-exposed individuals, respectively.

**Table 1:** Participants’ demographic and neuropsychological data

	TEC	PTSD	T / χ^2^	*p*
Participants	68	70		
Age (years)	44.44 ± 7.16	46.07 ± 6.40	-1.35	0.178
Gender(M/F)	23/45	17/53	1.52	0.217
CAPS^a^	9.28 ± 8.46	70.94 ± 21.56	-22.10	< 0.01
HAMA	3.79 ± 4.09	15.26 ± 7.87	-10.78	< 0.01
HAMD	3.38 ± 2.97	12.5 ± 6.10	-11.20	< 0.01
Mean FD	0.19 ± 0.09	0.20 ± 0.09	-0.301	0.764

Abbreviations: TEC, trauma-exposed control; PTSD, post-traumatic stress disorder; CAPS, clinician-administered PTSD scale; HAMA, Hamilton Anxiety Scale; HAMD, Hamilton Depression Scale

Statistical comparisons, gender, χ2 statistics; age, CAPS, HAMA, HAMD, t-tests

^a^Data are based on 68 TEC and 69 PTSD subjects, respectively

### ICN results

**Fig. 1:**
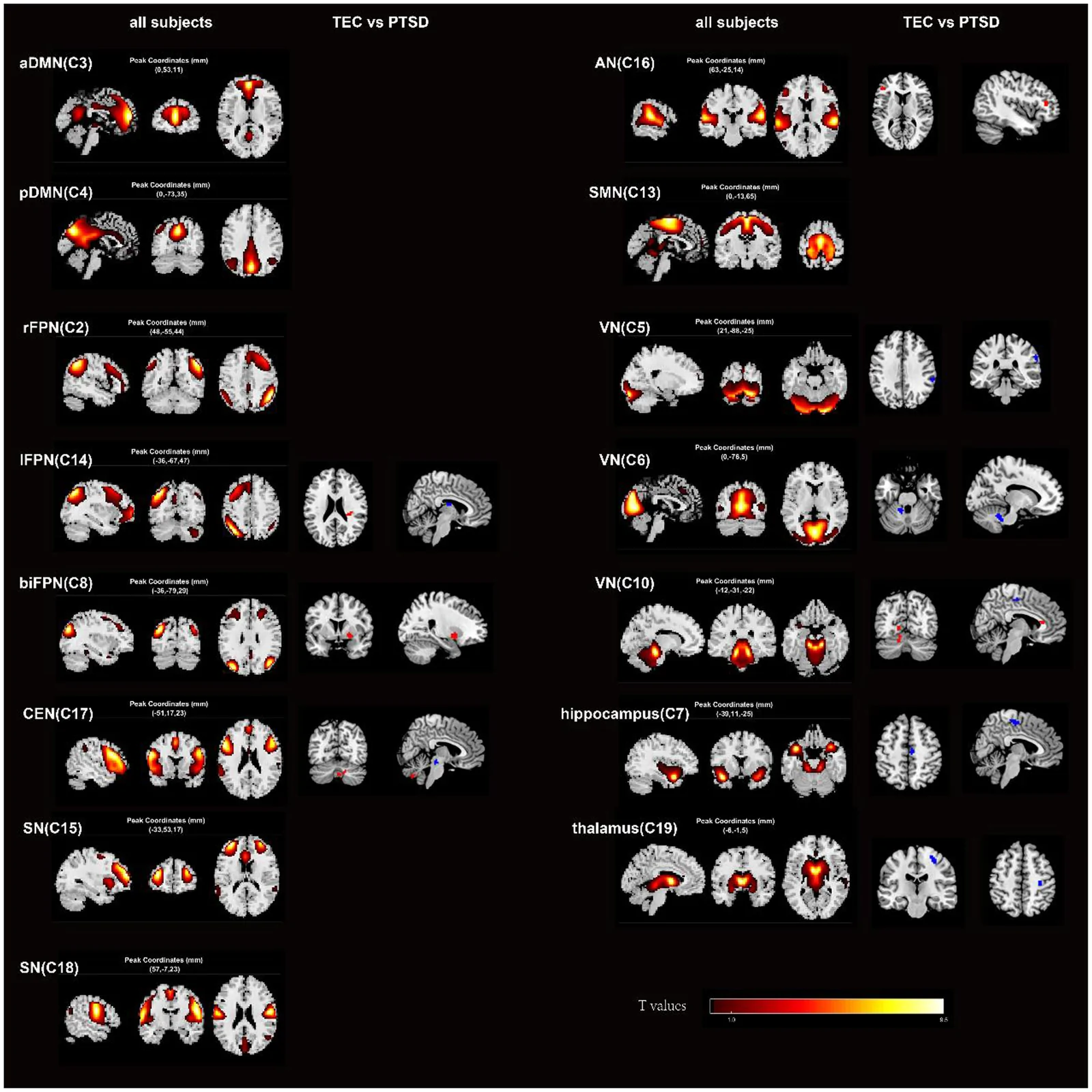
Spatial maps of 15 identified independent components (ICs)

A total of 20 independent components were initially estimated. The components were numbered according to their original output sequence (C1–C20). Through a combination of spatial and spectral inspection, 15 meaningful independent components were identified and selected according to their spatial correlation coefficients between ICs and corresponding templates [24], while four components (C1, C9, C11, and C12) were excluded due to their identification as artifacts, such as cerebrospinal fluid pulsations, head motion residuals, or white matter signals. 15 meaningful ICNs include right frontoparietal network (rFPN, C2), anterior default mode network (aDMN, C3), posterior default mode network (pDMN, C4), visual network (VN, C5), visual network (VN, C6), hippocampus network (C7), bilateral frontoparietal network (biFPN, C8), visual network (VN, C10), sensorimotor network (SMN, C13), left frontoparietal network (lFPN, C14), salience network (SN, C15), auditory network (AN, C16), cognitive executive network (CEN, C17), salience network (SN, C18), thalamus network (C19) (Fig. 1). These RSNs were organized in 8 groups: DMN (C3, C4), FPN (C2, C8, C14), CEN (C17), SN (C15, C18), AN(C16), SMN (C13), VN (C5, C6, C10), Subcortex Network (SC, C7, C19) (Fig. 1). Subsequent post-processing was the application of a fifth-order Butterworth band-pass filter (0.01–0.15 Hz) on the time-courses of the 15 independent components.

### ICN results and relevance with PTSD severity

For group comparisons, our analysis used independent-samples t-tests. Age and sex were included as nuisance covariates in all models. After employing a cluster-defining threshold (CDT) of *P* < 0.001, followed by cluster-level FWE correction (*P* < 0.05). Our results revealed that PTSD patients exhibited extensive variations in the functional connectivity of intrinsic brain networks across cortical (lFPN, biFPN, CEN, AN, VN) and subcortical (hippocampus and thalamus) networks. In comparison to TECs, we observed both reductions and enhancements in functional connectivity mainly in lFPN (C14), CEN (C17) and VN (C10). In PTSD patients, increased intrinsic functional connectivity (iFC) was found in biFPN (C8) and AN (C16) whereas diminished iFC was noted in visual network (C5 and C6). Furthermore, subcortical iFC was significantly reduced in hippocampal and thalamic networks (Fig. 1; Table 2).

**Table 2:** Difference of functional connectivity of the brain regions (PTSD vs. TEC group) in each intrinsic network connectivity (ICN)

ICN	Brain regions	Cluster Size(voxel)	Peak t-score	MNI coordinates		
				x	y	z
IFPN(C14)	thalamus	31	-4.03	-9	-3	18
	caudate nucleus (R)	21	4.41	27	-30	27
biFPN(C8)	putamen(R)	36	4.13	21	3	-6
CEN(C17)	Brain stem	28	-4.21	-3	-24	-15
	Cerebellum(R)	36	4.48	3	-72	-42
AN(C16)	IFG(L)	26	4.14	-42	33	12
VN(C5)	Angular gyrus(R)	25	-4.14	60	-36	36
VN(C6)	Cerebellum(L)	26	-4.61	-12	-45	-27
VN(C10)	MFG(R)	20	-4.03	9	-21	51
	Precentral gyrus(R)	24	-3.51	33	-15	51
Hippocampus(C7)	cuneus(R)	46	-4.52	18	-72	12
	MFG(R)	26	-4.23	6	-18	57
Thalamus(C19)	Precentral gyrus(R)	35	-4.52	30	-24	54

Abbreviations: PTSD, post-traumatic stress disorder; TEC, trauma-exposed control; medial frontal gyrus; lFPN, left frontoparietal network; biFPN, bilateral frontoparietal network; VN, visual network; IFG, inferior frontal gyrus; MFG, middle frontal gyrus

**Fig. 2:**
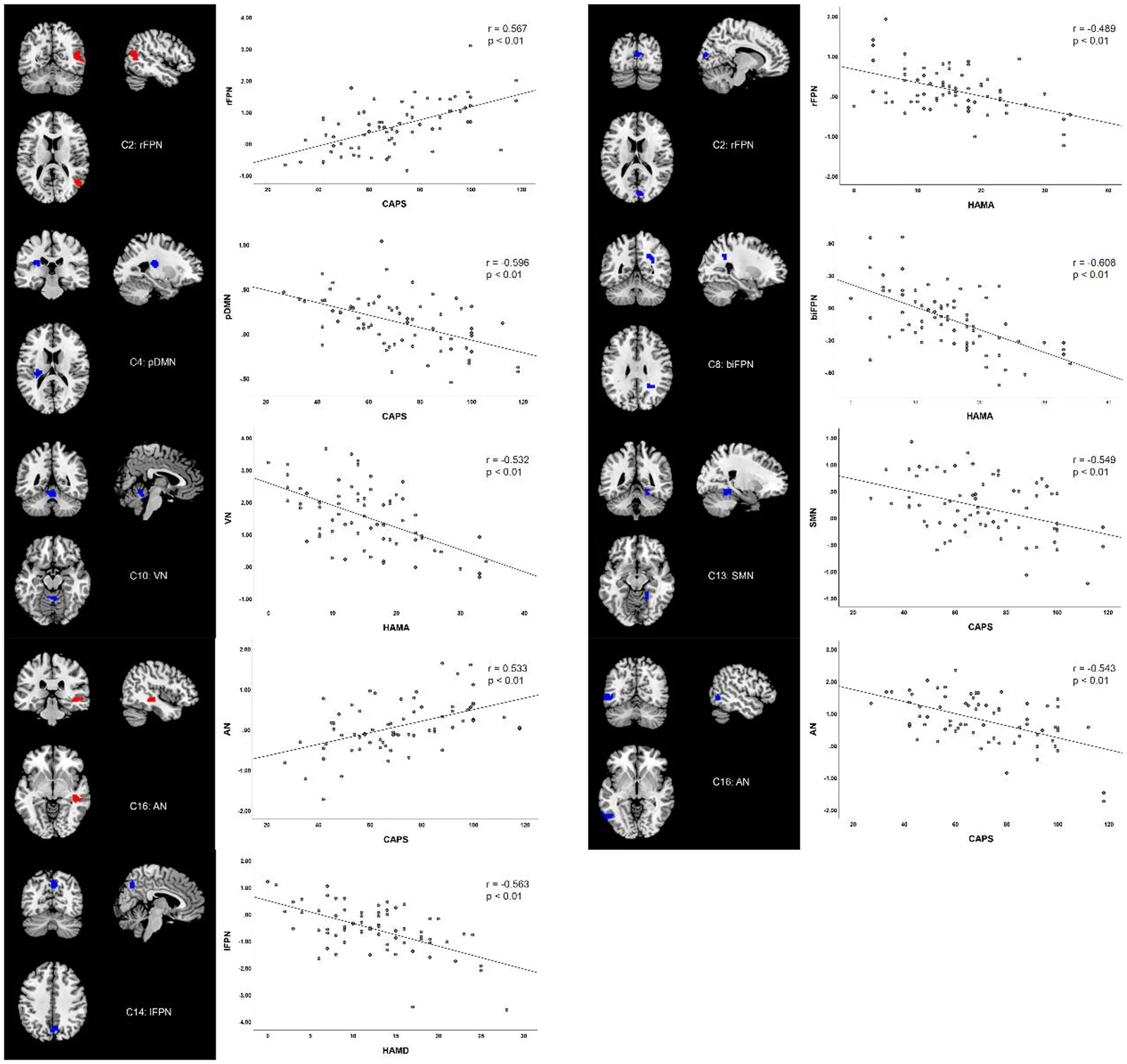
Brain regions showing significant correlations between ICN and clinical severity (CAPS, HAMD, HAMA) (cluster-level *P* < 0.05, FWE corrected). Scatter plots show the partial correlations between the mean ICN of the significant clusters and the CAPS, HAMD, HAMA scores. Abbreviations: ICN, intrinsic network connectivity; CAPS, Clinician-Administered Posttraumatic Stress Disorder Scale; HAMD, the Hamilton Rating Scale for Depression; HAMA, the Hamilton Rating Scale for Anxiety

In the voxel-wise ICN analysis by employing a cluster-defining threshold (CDT) of *P* < 0.001, followed by cluster-level FWE correction (*P* < 0.05), significant positive correlations were demonstrated between the CAPS scores and the rFPN(C2) in the right middle temporal gyrus (MTG) as well as AN(C16) in the right MTG. Conversely we identified substantial negative relationships between the CAPS scores and the pDMN(C4) in left internal capsule, SMN(C13) in the right cerebellum, and AN(C16) in the left MTG (cluster-level *P* < 0.05, FWE corrected). In addition, significant negative correlations between the HAMA scores and the rFPN(C2), biFPN(C8), and VN(C10) in the right cuneus, left parietal lobe and cerebellum were discovered respectively (cluster-level *P* < 0.05, FWE corrected). It is also important to note that HAMD scores were only negatively correlated with lFPN(C14) in the right precuneus (cluster-level *P* < 0.05, FWE corrected). To account for the potential confounding variables such as age and gender, we performed partial correlation analyses while controlling for these additional covariates. The detailed results, including MNI coordinates of peak activations and their corresponding t- and p-values, were presented in Table 3. (Fig. 2; Table 3). Mean connectivity values were extracted from the significant clusters identified in the primary voxel-wise GLM (encompassing both positive and negative findings) for each participant in the PTSD group. These values were then correlated with clinical symptom severity—measured by CAPS, HAMA, and HAMD scores using Pearson’s correlation. We presented the resulting scatter plots and correlation coefficients solely for descriptive and visualization purposes to illustrate the nature of the associations within the pre-identified networks. It is acknowledged that these clusters were pre-selected based on the whole-brain analysis; therefore, these post-hoc correlations are intended to clarify clinical trends rather than for independent statistical inference.

**Table 3:** Brain regions in ICN showing correlations with PTSD symptoms

ICN	Clinical variables	Brain regions	Cluster Size(voxel)	Peak t-score	MNI coordinates		
					x	y	z
rFPN(C2)	CAPS	MTG(R)	94	4.57	48	-66	18
rFPN(C2)	HAMA	cuneus(R)	49	-4.41	9	-84	15
pDMN(C4)	CAPS	internal capsule (L)	67	5.99	-27	-24	21
biFPN(C8)	HAMA	parietal lobe	53	4.55	33	-48	21
VN(C10)	HAMA	cerebellum	52	4.60	6	-51	-15
SMN(C13)	CAPS	cerebellum	47	4.79	24	-48	-18
lFPN(C14)	HAMD	precuneus(R)	55	4.84	6	-66	39
AN(C16)	CAPS	MTG(R)	49	4.73	42	-27	-6
AN(C16)	CAPS	MTG(L)	61	-5.09	-54	-63	0

Abbreviations: ICN, intrinsic network connectivity; rFPN, right frontoparietal network; MTG, Middle temporal gyrs; pDMN, posterior default mode network; biFPN, bilateral frontoparietal network; VN, visual network; SMN, sensorimotor network; lFPN, left frontoparietal network; AN, auditory network; PTSD, post-traumatic stress disorder; CAPS, clinician-administered PTSD scale; HAMA, Hamilton Anxiety Scale; HAMD, Hamilton Depression Scale

### dFNC results

Since the occurrence of dFNC states varied across participants, demographic and neuropsychological variables were therefore analyzed and reported on a state-wise basis. The total number of subjects on each state varies and is less than the 138 available subjects. Distribution of subjects among dFNC states, as well as state-wise demographic information, were displayed in Table 4. There were no significant differences in age between the TEC and PTSD groups in each state. Gender in State 4 and State 6 exhibited significant group difference (*p* < 0.05). Significant differences in clinical severity between PTSD and TEC groups were observed across all dFNC states.

**Table 4:** Demographics per dFNC states

Participants in each dFNC-state	TEC	PTSD	t	*p*
State 1	22	23		
Age (years)	45.68 ± 6.74	46.43 ± 6.38	-0.39	0.702
Gender(M/F)	10/12	15/8	1.78	0.16
CAPS	7.91 ± 7.21	69.91 ± 18.74	-14.77	< 0.01
HAMA	3.64 ± 4.02	15.22 ± 5.90	-7.66	< 0.01
HAMD	3.14 ± 2.12	12.74 ± 5.07	-8.36	< 0.01
State 2	15	24		
Age (years)	47.93 ± 4.51	48.79 ± 5.82	-0.49	0.63
Gender(M/F)	8/7	8/15	1.28	0.26
CAPS	11.87 ± 9.65	67.96 ± 21.65	-11.06	< 0.01
HAMA	4.73 ± 5.34	15.25 ± 9.40	-4.45	< 0.01
HAMD	4.67 ± 2.64	13.08 ± 7.31	-5.13	< 0.01
State 3	51	52		
Age (years)	44.16 ± 8.62	46.19 ± 6.48	-1.357	0.178
Gender(M/F)	15/36	12/40	0.534	0.465
CAPS	9.84 ± 9.02	72.88 ± 22.87	-18.31	< 0.01
HAMA	3.96 ± 4.35	15.73 ± 8.30	-9.04	< 0.01
HAMD	3.47 ± 3.03	12.85 ± 6.50	-9.41	< 0.01
State 4	25	32		
Age (years)	42.88 ± 8.93	43.25 ± 6.31	-0.18	0.86
Gender(M/F)	10/15	2/30	0.62	0.002
CAPS	10.36 ± 9.84	69.10 ± 18.68	-15.10	< 0.01
HAMA	4.12 ± 5.05	14.78 ± 7.48	-6.12	< 0.01
HAMD	3.40 ± 3.78	12.56 ± 6.0	-6.67	< 0.01
State 5	38	36		
Age (years)	45.82 ± 6.25	44.14 ± 5.49	1.22	0.346
Gender(M/F)	15/23	7/29	3.55	0.06
CAPS	8.87 ± 7.49	71.94 ± 23.97	-14.91	< 0.01
HAMA	4.24 ± 4.80	14.83 ± 7.36	-7.30	< 0.01
HAMD	3.42 ± 3.29	11.69 ± 5.47	-7.85	< 0.01
State 6	30	33		
Age (years)	47.33 ± 5.70	46.09 ± 6.75	0.791	0.432
Gender(M/F)	14/16	5/28	7.41	0.006
CAPS	10.07 ± 9.52	70.84 ± 22.62	-13.94	< 0.01
HAMA	4.13 ± 3.98	14.55 ± 5.85	-8.33	< 0.01
HAMD	3.73 ± 3.12	13.06 ± 4.98	-8.99	< 0.01
State 7	39	40		
Age (years)	43.74 ± 7.58	45.33 ± 6.41	-1.00	0.319
Gender(M/F)	15/24	8/32	3.26	0.07
CAPS	9.90 ± 8.76	71.45 ± 22.04	-16.39	< 0.01
HAMA	3.72 ± 4.19	14.58 ± 7.85	-7.69	< 0.01
HAMD	3.74 ± 3.32	11.98 ± 5.83	-7.74	< 0.01

Abbreviations: dFNC, Dynamic functional network connectivity; TEC, trauma-exposed control; CAPS, clinician-administered PTSD scale; HAMA, Hamilton Anxiety Scale; HAMD, Hamilton Depression Scale

**Fig. 3:**
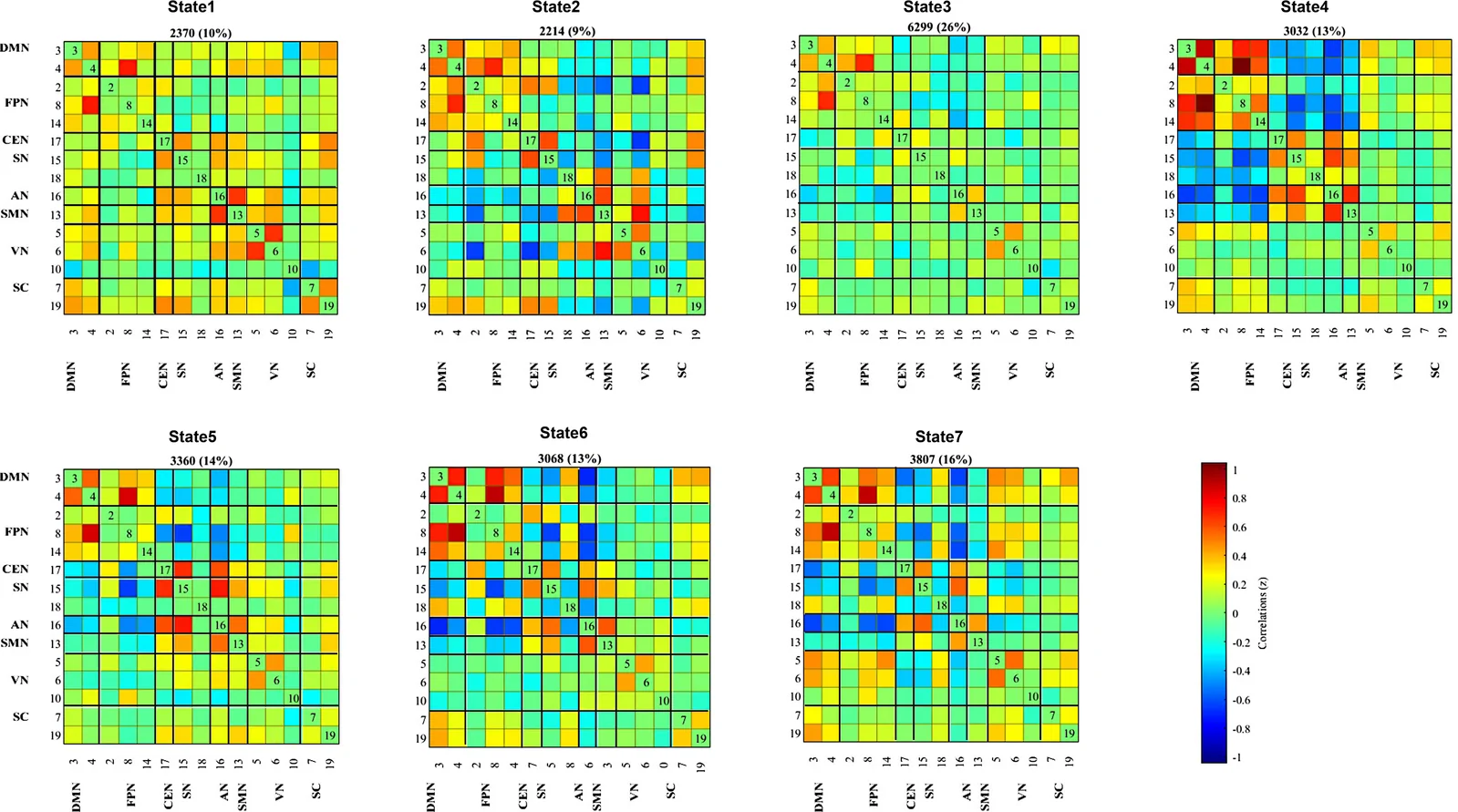
Seven dFNC states were identified across subjects. For each state, the dFNC matrix indicates the correlation between each IC pair. The order of ICs within each network is as follows: aDMN, pDMN, rFPN, biFPN, CEN, SN, AN, SMN, VN, SC (hippocampus and thalamus). The percentages of total occurrences are listed above each cluster. dFNC dynamic functional network connectivity, IC independent component, aDMN anterior default mode network, pDMN posterior default mode network, rFPN right frontoparietal network, biFPN bilateral frontoparietal network, CEN cognitive executive network, SN salience network, AN auditory network, SMN sensorimotor network, VN visual network, SC subcortical network

Seven dFNC states were discerned in the k-means clustering analysis (Fig. 3). The occurrence frequencies for states 1–7 were 10%, 9%, 26%, 13%, 14%, 13% and 16%, respectively. State 1 was involved by 22 trauma-exposed controls and 23 PTSD patients, state 2 was observed in 15 TEC and 24 PTSD subjects, state 3 involved 51 TEC and 52 PTSD participants, state 4 included 25 TEC and 32 PTSD individuals, state 5 was composed of 38 TEC and 36 PTSD subjects, state 6 was engaged by 30 TEC and 33 PTSD persons, state 7 comprised 39 TEC and 40 PTSD people.

Two sample t-tests were conducted on the mean dFNC correlations across the seven states. The mean dFNC matrices for each group (TEC and PTSD), as well as the t-value matrices, were provided in the supplementary material. The p-values were adjusted utilizing the false discovery rate (FDR). Significant group differences were observed in all states except State 4. In state1, increased dFNC anomalies were observed in the rFPN(C2) and hippocampus(C7) when compared to the TEC group. In state2, increased dFNC aberrations were identified in the CEN(C17) with aDMN(C3) and lFPN(C14) with SN(C15), whereas decreased dFNC aberrations were noted in the VN(C6) and aDMN(C3) within the PTSD cohort. In State3, patients with PTSD showed widespread alterations in thalamic (C19) connectivity, with decreased connectivity between the thalamus(C19) and VN(C6), and increased connectivity between the thalamus and both CEN(C17) and the hippocampus (C7). Additionally, connectivity was decreased between SN(C15) - biFPN(C8), and between AN(C16) - VN(C10). In state5, PTSD patients exhibited weaker dFNC compared to TECs in the SN(C15) and VN(C5), as well as VN(C6) and thalamus(C19). In state6, elevated dFNC aberrances were revealed between the thalamus(C19) and hippocampus(C7), as well as between VN(C6) with VN(C5), while reduced dFNC aberrances were observed in the thalamus(C19) with VN(C6) in the PTSD group. In state7, compared with the TEC group, hyper-dFNC aberrances were demonstrated in the pDMN(C4) with VN(C5), SN(C18) with hippocampus(C7), whereas hypo-dFNC alterations were identified in the thalamus(C19) and VN(C6) in the PTSD group. The complete t-value and p-value matrices for all 15 × 15 network pairs in each dynamic state were provided in Supplementary Figure S3.

### Association between dFNC and clinical symptom severity

There was no statistical difference in temporal metrics, including FT, MDT and NT in all the states between the groups following false discovery rate (FDR) correction. Partial correlation analysis was performed between time-varying characteristics (FT, MDT, NT) and clinical scores (CAPS, HAMA, HAMD) of the PTSD patients after regressing out age and gender effects. No substantial relationships were identified between the dFNC matrix and clinical severity.

## Discussion

This study investigated both static and dynamic functional brain network connectivity in a well-characterized cohort of treatment-naïve adults with PTSD compared to trauma-exposed controls. By employing a data-driven independent component analysis (ICA) and a sliding-window approach, we identified significant alterations in iFC and dynamic functional network connectivity (dFNC). Our principal findings reveal that PTSD is characterized by: (1) widespread alterations in sFNC within cortical (FPN, DMN, AN, VN) and subcortical (hippocampus, thalamus) networks, with the magnitude of these changes correlating with clinical symptom severity; and (2) abnormal connectivity patterns within specific, recurrent dFNC states, particularly involving interactions between higher-order cognitive networks, low-level perceptual networks, and subcortical hubs. By demonstrating alterations in subcortical-cortical connectivity, our results support a systems-level model of PTSD, where impaired “top-down” regulation from the FPN is compounded by “bottom-up” sensory-limbic instability. These results extend our understanding of PTSD as a disorder of large-scale network dysregulation and highlight the value of a dynamic perspective in uncovering the moment-to-moment neural dysfunction underlying its complex symptomatology.

### Static network connectivity: a foundation of widespread dysregulation

Our analysis of static iFC revealed widespread abnormalities in PTSD, aligning with a substantial corpus of research suggesting that the disorder impacts distributed neural systems [7]. We observed significant alterations in the FPN, a key system for executive functions like attention control and emotion regulation. The correlation between FPN connectivity and the severity of PTSD, anxiety, and depression symptoms in our cohort may reflect the network’s potential involvement in the disorder’s neurobiological profile, suggesting an association between frontoparietal integrity and clinical symptom manifestation. This finding aligns with models suggesting that PTSD involves a failure of top-down cognitive control, mediated by the FPN, over prepotent emotional and memory-related processes [8]. The disruption of this network may prevent individuals from disengaging from trauma-related thoughts and effectively regulating their emotional responses.

A notable finding of our study is the significant alteration within low-level perceptual networks, including the visual (VN), auditory (AN), and sensorimotor (SMN) networks. These results provide a neural correlate for the pervasive sensory symptoms of PTSD, such as hypervigilance, exaggerated startle response, and intrusive sensory memories (e.g., flashbacks). Previous work has also identified abnormal connectivity in these networks in trauma survivors [17]. Our observation that connectivity within the SMN and AN was negatively correlated with CAPS scores suggests a complex relationship, where disruptions in sensory integration are intrinsically linked to overall symptom burden. This may reflect a state of disordered sensory gating, where the brain fails to properly filter and integrate incoming sensory information, leading to a state of constant arousal and perceptual distortion.

Furthermore, we confirmed the well-established involvement of subcortical structures in PTSD. Decreased iFC in the hippocampus and thalamus networks aligns with decades of research implicating these regions in the disorder. The hippocampus is critical for encoding contextual information and integrating memories into a coherent autobiographical narrative [25]. Its dysfunction in PTSD is thought to contribute to the fragmented, decontextualized nature of trauma memories that intrude into present awareness. The thalamus, as the brain’s primary sensory relay station, plays a crucial role in gating the flow of information to the cortex. Attenuated thalamic connectivity, as observed here and in other studies, may reflect impaired sensory processing, contributing to the overwhelming nature of sensory experiences in PTSD [26].

### Dynamic network connectivity: revealing aberrant brain states

While sFNC provides a foundational map of dysfunction, our dFNC analysis offers a more granular view of the moment-to-moment brain dynamics that are disrupted in PTSD. The central finding is that individuals with PTSD exhibit altered connectivity patterns within specific, recurring brain states, rather than a global change in temporal dynamics. This suggests that the disorder is characterized by an abnormal repertoire of functional configurations that the brain can adopt. Furthermore, the number of participants transitioning into each state varied, leading to unequal sample sizes for state-wise comparisons. While such imbalance can theoretically affect statistical power and robustness, our between-group comparisons of temporal metrics (fractional occupancy, mean dwell time, and number of transitions) across all seven dFNC states yielded no significant differences between the PTSD and TEC groups, nor any significant correlations with clinical scales. This is distinct from the dFNC connectivity pattern analyses, in which significant group differences were identified in six of the seven states (all except State 4). This suggests that the current findings are not driven by spurious, underpowered effects. However, the absence of significant dFNC alterations in this study should be interpreted within the context of our specific sample composition.

Across the seven dFNC states, the most consistent finding was widespread thalamic involvement, with the thalamus showing altered connectivity to CEN, hippocampus, and VN in States 3, 6, and 7. The opposing directions of these changes across states suggest the thalamus acts as a dynamically unstable hub, whose sensory gating and relay functions fluctuate with PTSD symptom states such as hyperarousal and intrusive re-experiencing. A second theme involves altered coupling among higher-order networks—SN, FPN, CEN, and DMN. Increased CEN-DMN and FPN-SN coupling in State2, alongside decreased SN-FPN coupling in States 3, suggest a blurring of normally segregated network boundaries (dedifferentiation), which may impair the brain’s ability to suppress trauma-related intrusions and flexibly direct attention between internal and external processing. Finally, recurrent alterations involving the hippocampus and visual networks (States 1, 3, 5, and 7) point to disrupted memory-sensory integration, potentially contributing to the vivid sensory quality of trauma-related flashbacks. This lack of statistical significance suggests that the functional configurations within State 4 may represent a relatively stable or ‘canonical’ dynamic mode that remains largely preserved in the aftermath of trauma, regardless of a PTSD diagnosis. Unlike the other states that exhibited disease-related dysregulation, State 4 might reflect a shared neural baseline or a common functional scaffold between TEC and PTSD groups.

Interestingly, no significant group differences were observed in the temporal properties of these states, such as the FT spent in each state, the MDT, or the NT. This null result aligns with the findings of the large-scale ENIGMA-PGC study, which also failed to find robust differences in these dFNC metrics [7]. In contrast, studies in pediatric PTSD have reported reduced network switching rates, suggesting a more rigid network configuration [8]. Taken together, these findings suggest that the core dynamic pathology in adult PTSD may lie more in the nature of the accessible brain states (i.e., the specific patterns of connectivity) rather than in the overall temporal flow between them. The brain may transition between states at a normal rate, but the states themselves are dysfunctional. In contrast to the significant associations found between ICN voxel-wise measures and clinical severity, our analysis of dFNC, including temporal metrics (NT, MDT, FT) and state-specific matrices—yielded no substantial relationships with clinical scales (all *P* > 0.05). These negative findings suggest that while spatial functional integrity is linked to CAPS and HAMA, the short-term temporal dynamics of these networks may remain relatively independent of symptom severity in the current cohort.

### Clinical implications and future directions

Our findings have several important clinical implications. The confirmation of widespread static and dynamic network abnormalities reinforces the view of PTSD as a brain network disorder, suggesting that effective treatments must target this distributed dysregulation. Future research is warranted to explore whether the specific networks implicated in our study—namely the FPN, could potentially serve as candidates for neuromodulatory interventions, such as transcranial magnetic stimulation (TMS) or neurofeedback. The dFNC results, in particular, suggest that a key therapeutic goal might be to restore more adaptive brain states and promote flexible integration between cognitive control and sensory processing networks.

The correlations between iFC and symptom severity also highlight the potential of these measures as biomarkers for diagnosis, prognosis, or tracking treatment response [27, 28]. Future research could use machine learning approaches to determine if dFNC state patterns can predict which individuals are most at risk for developing PTSD after trauma or which patients will respond best to specific therapies.

### Limitations

This study has several limitations. First, the cross-sectional design precludes causal inferences regarding whether the observed connectivity abnormalities precede or result from PTSD; longitudinal studies tracking individuals from pre-trauma through treatment are needed to clarify these pathways. Second, although our sample was well-characterized and homogeneous in treatment status and comorbidities, it was of modest size for dFNC analysis, and the subset of participants transitioning into State 4 and State 6 showed significant sex imbalances despite overall sex-matching between groups; larger, multi-site replications with attention to sex-specific connectivity signatures are warranted. Third, our focus on a single trauma type (earthquake) enhances internal validity but may limit generalizability to PTSD arising from other traumas such as combat or interpersonal violence [2]. Finally, dFNC results are inherently sensitive to methodological choices (e.g., sliding window length, number of clusters) and data composition, despite our use of a multi-iteration k-means approach (150 repetitions) to ensure clustering stability.

A related concern involves subclinical affective burden. Although all PTSD participants were free of comorbid MDD/GAD by SCID-5, mean HAMD and HAMA scores (12.5 and 15.26) indicate non-trivial subclinical symptoms that were only moderately correlated with CAPS severity (*r* = 0.483 and 0.452) (Supplementary Table S2). Sensitivity analyses controlling for HAMD/HAMA revealed a dissociation: CAPS-ICN regression findings remained largely robust (3 of 4 ICNs survived) (Supplementary Figure S2, Table S4) whereas between-group differences attenuated markedly (2 of 9 ICNs survived) (Supplementary Figure S1, Table S3). While over-adjustment bias and multicollinearity-related power loss (*N* = 69–70) likely contributed, this attenuation suggests that some between-group differences may partly reflect transdiagnostic affective processes rather than PTSD-specific neural reorganization. The between-group connectivity findings should therefore be interpreted with caution, pending replication in larger samples that explicitly model affective symptom burden.

## Conclusion

In conclusion, the results of this study provide insights into the static and dynamic organizational properties of brain networks in PTSD, implying that network-level signatures could be potentially linked to the neural mechanisms underlying the condition in adults. The findings at the global and subnetwork levels, highlighted the role of frontoparietal, visual, as well as subcortical networks. The observed network specificity supports the view that PTSD is characterized by disrupted network integration, impaired attention and perceptual processing. Considering the dynamic nature of brain activity and connectivity, we would like to emphasize the significant potential to explore the underlying neural pathophysiology of enduring mental health symptoms in PTSD survivors through the time-varying properties of dFNC. These findings move beyond a static view of the disorder, offering a more nuanced model where the inability to achieve adaptive modes of network integration underlies the persistent and complex symptoms of PTSD. The dynamic approach holds significant promise for identifying novel biomarkers and developing targeted interventions aimed at restoring flexible and adaptive brain function in those who have survived trauma.

## Supplementary Information

Below is the link to the electronic supplementary material.

Supplementary Material 1

## Data Availability

The data that support the findings of the current study are available from the corresponding authors upon reasonable request.

## Abbreviations

PTSD: Post-traumatic stress disorder
TECs: Trauma-exposed controls
dFNC: dynamic functional network connectivity
rs-fMRI: resting-state functional magnetic resonance imaging
CAPS: Clinician-Administered post-traumatic stress disorder Scale
HAMA: The Hamilton Anxiety Rating Scale
HAMD: Hamilton Depression Rating Scale
FPN: Frontoparietal network
DMN: Default mode network
VN: Visual network
SMN: Sensorimotor network
SN: Salience network
AN: Auditory network
CEN: Cognitive executive network
