# Supplementary material for: Exploring the spatio-temporal functional connectome in PTSD: evidence of static and dynamic network alterations
Source: BMC Psychiatry. 2026 Aug 6;26:865. doi: 10.1186/s12888-026-08500-4 (PMC13732884; doi:10.1186/s12888-026-08500-4)
Supplement: Supplementary file 1 [file 12888_2026_8500_MOESM1_ESM.docx]

**Methods:**

**Voxel-wise ICN–symptom tests**

To investigate the relationship between functional connectivity (FC) measures and clinical symptom severity, we employed a mass-univariate multiple regression analysis (also referred to as the General Linear Model, GLM) across all PTSD participants. The detailed statistical framework was as follows:

For each intrinsic connectivity network (ICN) or functional edge, a multiple regression model was constructed where the voxel-wise connectivity strength served as the dependent variable (y). The primary variable of interest was the clinical scale score (e.g., CAPS-5, HAMD, or HAMA). To ensure the specificity of the findings, age and sex were included in the model as nuisance covariates to regress out their potential confounding effects on brain connectivity. In line with established protocols[1, 2], we defined specific t-contrasts to test for directionality. For the positive association: a contrast vector of [0, 0, 1] (assuming covariates were the first two columns) was applied to identify regions where connectivity strength increased linearly with symptom severity. For the negative association: a contrast vector of [0, 0, -1] was applied to identify regions where connectivity strength decreased as symptoms intensified. Statistical significance was determined using cluster-level FWE correction with a cluster-defining threshold of P < 0.001. This methodology allows for a robust identification of the neural substrates that uniquely contribute to clinical phenotypes while maintaining strict control over Type I errors.

**State-wise dFNC edge tests**

For each of the seven identified states, two-sample t-tests were conducted to compare the functional connectivity strength (edges) between the PTSD and control groups. With 15 independent component networks (ICNs) identified, a total of 105 unique pairwise edges were tested within each state. Multiple comparisons were controlled using the False Discovery Rate (FDR) method (p< 0.05) across these 105 edges (15 x (15-1)/2 = 105). While FDR was applied to the 105 edges within each state to maintain local statistical rigor, the findings across the seven states were interpreted with caution, acknowledging the exploratory nature of this state-wise analysis.

**Temporal metrics comparisons**

Three core temporal metrics were calculated for each participant: Fractional Occupancy (FT) (the proportion of time spent in each state), Mean Dwelling Time (MDT) (the average duration of staying in a state before transitioning), and Number of Transitions (NT) (the total frequency of switching between different states). Two-sample t-tests (PTSD vs. TEC) were performed to assess group differences in these metrics. For state-specific metrics (FT and MDT), FDR correction was applied across a family of 14 tests (7 states x 2 metrics) using the GRETNA toolbox. For the global metric (NT), since it is a single value per subject representing overall dynamics, it was tested separately. All results were FDR-corrected p<0.05.

**Results**

**Table S1.** Effect sizes for group differences in temporal metrics (FT, MDT, and NT)

| dFNC of PTSD | Cohen’s d |
| --- | --- |
| NT | 0.182 |
| MDT1 | -0.032 |
| MDT2 | 0.229 |
| MDT3 | -0.236 |
| MDT4 | 0.051 |
| MDT5 | -0.197 |
| MDT6 | 0.091 |
| MDT7 | 0.013 |
| FT1 | 0.034 |
| FT2 | 0.273 |
| FT3 | -0.212 |
| FT4 | 0.044 |
| FT5 | -0.174 |
| FT6 | 0.12 |
| FT7 | -0.01 |

**Sensitivity analysis results:**

**Table S2.** Spearman correlations among CAPS, HAMA, and HAMD scores in PTSD patients (N = 69-70)

|  |  | **CAPS** | **HAMA** | **HAMD** |
| --- | --- | --- | --- | --- |
| **CAPS** | r | 1.000 | **.452**** | **.483**** |
|  | p | – | <.001 | <.001 |
|  | N | 69 | 69 | 69 |
| **HAMA** | r | **.452**** | 1.000 | **.787**** |
|  | p | <.001 | – | <.001 |
|  | N | 69 | 70 | 70 |
| **HAMD** | r | **.483**** | **.787**** | 1.000 |
|  | p | <.001 | <.001 | – |
|  | N | 69 | 70 | 70 |
| ***Correlation is significant at the 0.01 level (2-tailed).* | | | | |

**Two-sample t-tests (PTSD N = 70 vs. TEC N = 68):** In the primary analysis (covariates: age, gender), 9 ICNs showed significant between-group differences across 13 clusters. When HAMD and HAMA were additionally entered as nuisance covariates, only 2 ICN (C10, VN) and (C19, Thalamus) survived, representing a substantial attenuation of the original findings.

**Figure S1**. Group differences in static functional network connectivity between PTSD and TEC, before and after adjusting for depressive and anxiety symptoms.

**Table S3** Difference of functional connectivity of the brain regions (PTSD vs TEC group) in each intrinsic network connectivity (ICN) after sensitivity analysis (adjusting for HAMA and HAMD).

| ICN | brain  regions | cluster  Size(voxel) | peak  t-score | MNI coordinates | | |
| --- | --- | --- | --- | --- | --- | --- |
|  |  |  |  | x | y | z |
| VN(C10) | premotor cortex and supplementary motor areas (R) | 88 | -4.77 | 27 | -18 | 51 |
| Thalamus(C19) | Precentral gyrus(R) | 24 | -4.52 | 33 | -24 | 54 |

**CAPS-ICN regression (PTSD N = 69):** In the primary analysis (covariates: age, gender), 4 ICNs showed significant associations with CAPS severity across 5 clusters. When HAMD and HAMA were additionally entered as covariates, 3 of 4 ICNs (75%) remained significant with largely consistent spatial distributions, whereas the association involving AN (C16) did not survive correction in one of its regional foci (Supplementary Figure S2, Table S3).

**Figure S2** Associations between ICN connectivity and CAPS severity in PTSD patients, before and after adjusting for depressive and anxiety symptoms.

**Table S4** Brain regions in ICN showing correlations with CAPS after sensitivity analysis (adjusting for HAMA and HAMD).

| ICN | clinical  variables | brain  regions | cluster  Size(voxel) | peak  t-score | MNI coordinates | | |
| --- | --- | --- | --- | --- | --- | --- | --- |
|  |  |  |  |  | x | y | z |
| rFPN(C2) | CAPS | MTG(R) | 69 | 4.22 | 48 | -66 | 18 |
| pDMN(C4) | CAPS | internal capsule (L) | 48 | 5.26 | -27 | -21 | 18 |
| AN(C16) | CAPS | MTG(L) | 53 | -5.09 | -60 | -63 | 0 |

Abbreviations: ICN, intrinsic network connectivity; rFPN, right frontoparietal network; MTG, Middle temporal gyrs; pDMN, posterior default mode network; AN, auditory network; CAPS, clinician-administered PTSD scale; HAMA, Hamilton Anxiety Scale; HAMD, Hamilton Depression Scale.

**Figure S3.** Summary of statistical matrices (t-values and p-values) of functional connectivity among 15 brain networks across the seven dynamic states.

**Reference**

1.	Zhuo C, Wang C, Wang L, Guo X, Xu Q, Liu Y, Zhu J: **Altered resting-state functional connectivity of the cerebellum in schizophrenia**. *Brain imaging and behavior* 2018, **12**(2):383-389.

2.	Yang Y, Wang Y, Zhang C, Zhu J, Yu Y: **Neuroanatomical substrates underlying contrast sensitivity**. *Quantitative imaging in medicine and surgery* 2019, **9**(3):503-509.
